# Perceptions of Sudanese women of reproductive age toward HIV/AIDS and services for Prevention of Mother-to-Child Transmission of HIV

**DOI:** 10.1186/s12889-015-2054-1

**Published:** 2015-07-17

**Authors:** Ibrahim E. Elsheikh, Rik Crutzen, H.W. Van den Borne

**Affiliations:** Department of Health Promotion, CAPHRI School for Public Health and Primary Care, Maastricht University, Maastricht, The Netherlands; Sudanese Public Health Association (SPHA), Khartoum, Sudan

**Keywords:** HIV/AIDS, PMTCT, Pregnant women

## Abstract

**Background:**

Access to antenatal HIV testing during pregnancy and the level of uptake of services for Prevention of Mother-to-Child Transmission (PMTCT) in Sudan are very low. This study aimed to obtain insights into the perceptions of Sudanese pregnant women toward HIV/AIDS and the use of PMTCT services.

**Methods:**

Ten focus group discussions (FGDs) with women of reproductive age were conducted at community settings in Khartoum (*N* = 121). Recruitment eligibility included living near or around a PMTCT site and being in the age range of 18–40 years. Out of 121 women who participated, 72 (61 %) were pregnant. Predefined themes were addressed in the theory-based interview scheme, which was derived from multiple socio-cognitive theories—i.e., the Extended Parallel Process Model, the Reasoned Action Approach and the socio-psychological view on stigma. Emerging themes were incorporated during data analysis.

**Results:**

Few women knew about the Mother to child transmission (MTCT) of HIV. No one indicated that MTCT might occur during labor. Most women believed that HIV/AIDS is a serious and fatal condition for them and also for their children. They believed they were susceptible to HIV/AIDS as a result of cesarean section, contaminated items (blood and sharp items) and husband infidelity. The usefulness and advantages of HIV testing were questioned; for some women it was perceived as an additional burden of anxiety and worry. Doctors were the most influential with regard to acceptance of HIV testing. The speed of the testing process and confidentiality were mentioned by some women as key factors affecting willingness to undergo HIV testing at a health facility during pregnancy.

**Conclusion:**

The study reveals that most of the women felt susceptible to HIV infection with perceived high severity; however, this perception has not translated into positive attitudes toward the importance of HIV testing during pregnancy. Because of anticipated stigma, women are not likely to disclose their HIV status. Further research should focus on gaining a more in-depth understanding of the psycho-social determinants and processes underlying the factors identified above. In addition, the adequate implementation of Provider Initiated Testing and Counseling (PITC) should be critically assessed in future research about PMTCT in Sudan.

## Background

Every day, approximately 1500 children become infected with HIV worldwide [1]. More than 90 % of children living with HIV have been infected through mother-to-child transmission (MTCT) [2, 3]. Most of these infections, which occur largely in Sub-Saharan Africa [4], can be prevented by currently available interventions, such as HIV testing, enrollment in treatment from as early as 14 weeks of gestation (second trimester) [5], management of labor (Caesarean section), informed decisions on breastfeeding and pediatric care [6, 7]. These interventions are referred to as PMTCT services. There were 2300 [Lower estimate 1500–3400 higher estimate] new HIV infections among children in the Middle East and North Africa in 2013 [8].

In Sudan, the HIV epidemic is driven by heterosexual transmission, and a review conducted in 2010 estimated that out of all new HIV infection cases, 59 % occurred in women of reproductive age (15–49 years). PMTCT services are therefore a high priority. Effective delivery of PMTCT services is highly rewarding, as it can reduce the risk of MTCT of HIV infection and ultimately eliminate new HIV cases among children [7].

In Sudan, PMTCT services began in 2005 as a pilot in a few states. In 2007, there were seven sites; this was scaled up to more than 27 sites in 2010 [9] and 227 in 2013 [10]. The opt-out strategy was piloted in Sudan in 2007 and adopted in 2009; in 2012, Provider Initiated Testing and Counseling (PITC) was introduced. Although PMTCT services are provided through tertiary, secondary and primary levels of health care, full implementation of the standard PMTCT package is limited to the tertiary level [10]. Despite the potential impact that PMTCT can have on reducing and eliminating new HIV cases among children, access to antenatal HIV testing during pregnancy is very low (<1 %), as is access to antiretroviral therapy (<10 %) [7]. Moreover, the level of PMTCT uptake in Sudan is very low. In 2010, 70 % of pregnant women (or 108,588) attended antenatal care at 27 UNICEF-supported sites, but only 13 % (or 14,277) of these pregnant women were tested [9]. Sudan has adopted option B+ for the PMTCT treatment protocol since 2012 [10]. In option B+, women receive triple Antiretroviral (ARVs) starting as soon as they are diagnosed and continuing for life [11]. In 2013, only 7 % of pregnant women took an HIV test, mainly due to inefficient implementation of PITC, which can be attributed to health system factors such as the reluctance of health care providers to provide HIV testing for pregnant women, lack of accountability and weak integration of HIV testing as part of the routine check-ups for pregnant women [10]. Incomplete implementation of the opt-out strategy regarding HIV testing, limited training of health care providers and delay in shifting from vertical to integrated programs were also mentioned among key factors affecting the scale-up of PMTCT services [9, 7].

Very limited research about PMTCT has been done in Sudan, and has focused on knowledge and attitudes towards HIV voluntary and counseling testing services among pregnant women [12] and descriptive research about maternal HIV infection [7]. In a systematic review of health system barriers and enablers, a wide range of key factors affected PMTCT and especially enrollment of women in ARV including long waiting times, transport costs, confidentiality, poor staff attitudes and fragmented service delivery platforms [13]. In another review, individual-level factors such as financial limitations and fear of stigma were identified [14]. Regarding organizational factors, a multi-level assessment study in Sub-Saharan African countries showed that increased workload for health and medical cadres was negatively associated with the acceptance and uptake of HIV testing [15]. In study in Khartoum, out of the 1005 pregnant women interviewed, 55.9 % (562) had basic information about the mother to child transmission [12]. However, the knowledge of pregnant women’s psycho-social determinants regarding HIV testing in the context of PMTCT in Sudan is still incomplete, whereas studies from other African countries have shown some contradictory findings.

A study in Kenya revealed patient-level factors, such as being of younger age (15–24 years compared to 25–39 years), rural residence and lower educational levels, to be associated with less acceptance of HIV testing during pregnancy [16]. A study in Nigeria indicated a high level of knowledge about HIV (90 %) but less knowledge about PMTCT (68 %) [17]. Another study in Nigeria [18] also revealed that younger women were less likely to use PMTCT services; however, no association was found between socioeconomic status and utilization of these services. In a study in North Uganda, HIV testing was higher for women with primary education compared to those with no formal education [19]. A study in Botswana indicated that living in an urban area, having knowledge about PMTCT, knowing someone who had received ARV therapy or PMTCT, and having a partner who had been tested for HIV were all significantly associated with higher test acceptance [20]. Stigma negatively impacts PMTCT service uptake and treatment adherence. In a study in Kenya [21], there was a strong association between stigma and non-disclosure of HIV positive status.

The aim of this qualitative study was to obtain insights into the perceptions of pregnant Sudanese women toward HIV/AIDS and the use of PMTCT services (mainly testing) and to explore the perceived changeable factors associated with use of services. This area is under-researched in Sudan. Understanding the factors associated with service use is essential to stimulate future quantitative research and to develop interventions aimed at increasing the utilization of available PMTCT services.

## Methods

Ten focus group discussions (FGDs) with women of reproductive age were conducted at community settings in Sudan’s capital, Khartoum, in 2013. The number of women per group varied, the minimum being nine and the maximum 15. The duration of the discussions was 45 min to one hour maximum. Two female researchers were present in all focus group interviews. They were local Non-governmental Organization (NGO) outreach staff who had previously worked on different HIV/AIDS projects, and they received one-day training on the research guide. They spoke the local language (Arabic), and their age range was 29–35 years. Women were intentionally recruited due to the sensitivity of the topic and also to help stimulate the discussion. One served as a discussion facilitator who guided the discussion until all questions were thoroughly answered, while the other took notes and checked whether all topics were covered.

### Recruitment of participants

Purposive and simple random techniques were used to select the respondents. Whereas women were purposively selected, the communities and households were randomly identified. Ten communities from three localities in Khartoum state (Khartoum, Omdurman and Bahri) were purposively selected based on the fact that they were around the five main PMTCT sites in greater Khartoum. The sampling frame was the total number of households in the 10 communities that were located near or around the five PMTCT sites. Consecutive numbers were assigned to each household, and then 121 households were randomly selected. Women of reproductive age were recruited (*N* = 121) by community agents/volunteers upon entering the randomly selected houses. All eligible women from each household were recruited. This process was followed until all 121 women were selected. Most of the women who were approached accepted to participate. Recruitment eligibility included living near or around a PMTCT site and being in the age range of 18–40 years. Those who were pregnant were given preference. HIV status was not part of the selection criteria. Women were told in advance that the discussions would be about HIV and AIDS. Most of the actual participants fell mainly into two age bands of 20–29 and 30–38 years. Women of similar ages participated in the same FGD. Of the 121 women who participated, 72 (61 %) were pregnant.

### Procedure

Verbal informed consent was obtained from the women before the FGDs started. The women were told that there was no need for names and that the information exchanged was purely for research purposes. However, the facilitator assigned the participants codes with which to identify individuals while they were talking. Emphasis was laid on the importance of everyone’s participation, and they were also told that there were no correct or wrong answers. The facilitators used an interview scheme to stimulate the discussion. Discussions were held in Arabic in a community setting rather than a health facility to ensure the natural flow of the discussion, as women felt more at ease in their respective community places than at a health facility. In addition, all health facilities had very limited spaces for such group discussions.

### Interview discussion guide

The main topics for the focus group discussions are summarized in Table 1. The discussion guide, which was in Arabic, was pre-tested among 10 pregnant women from similar areas and was found to be good, except that the participants had difficulty understanding the concept of HIV/AIDS severity. Then, an equivalent term from a local community dialect of Arabic was added to the severity questions and was found to be easily understood by the same group. The focus group discussion began with questions regarding general information about HIV/AIDS and PMTCT, and then the focus was directed toward an in-depth discussion of factors perceived to be associated with the acceptance of HIV testing during pregnancy. The factors that were put forward for discussion were derived from multiple socio-cognitive theories—i.e., the Extended Parallel Process Model [22] (perceived severity, perceived susceptibility and response efficacy), the Reasoned Action Approach [23] (attitude, subjective norms, perceived behavioral control) and the socio-psychological view on stigma [24] (personal responsibility and norm-violating behavior). Where there was an overlap between these theories (e.g., self-efficacy in the Extended Parallel Process model and perceived behavioral control in the Reasoned Action Approach), these factors were only discussed once for the sake of parsimony. For perceived severity, perceived susceptibility, response efficacy and personal responsibility, a distinction was made between how these factors concerned the mother and how they concerned the child.

**Table 1:** Interview discussion guide

Theme	Example of questions
Knowledge about HIV/AIDS and PMTCT transmission/prevention	In your opinion, how do people get infected with HIV?
	How do you think we prevent ourselves and our children from being infected with HIV?
	To what extent do you think that the HIV virus can be transmitted from mother to child, and how?
	How can mother to child transmission be prevented?
Perceived severity, perceived susceptibility, response efficacy	How severe do you think the consequences of HIV are for *you*? And why?
	How severe do you think the consequences of HIV are for *your child, and why*?
	How likely do you think it is that *you* will be affected by HIV, and why?
	How likely do you think it is that *your child* will be affected by HIV? Why?
	How useful is HIV testing during pregnancy for *you*? Why?
	How useful is HIV testing during pregnancy for *your child?* Why?
Attitude, subjective norms, perceived behavioral control	What do you see as the advantages of you taking an HIV test during pregnancy?
	What do you see as the disadvantages of you taking an HIV test during pregnancy?
	What else comes to mind when you think of HIV testing during pregnancy?
	Who would approve and think you should undergo an HIV test during pregnancy?
	Who would disapprove and think you should not take an HIV test during pregnancy?
	What are the factors/circumstances that would make it easy for you to take an HIV test during pregnancy?
	What are the factors/circumstances that would make it difficult for you take an HIV test during pregnancy?
Personal responsibility, norm-violating behavior	To what extent is a *mother* responsible for getting HIV? Why?
	To what extent is a *child* responsible for getting HIV infected and why?
	Which behaviors are, according to you, related to becoming HIV infected? Why?
	In your opinion: Do you think that a person who is HIV+ but not sick should be allowed to continue working with the public? (E.g. a teacher, a nurse or a doctor) Why?
	If someone is HIV+ should s/he let others know her/his status*?* Why*?*

### Data analysis

Audio tapes were not used for any of the FGDs because the participants were suspicious of the discussions being recorded. To overcome this shortfall, a note-taker who was moderator’s research assistant accompanied the facilitator during the FGDs. At the end of each FGD the note-taker and the moderator sat together to revise and summarize the data in the notebook. The notes were collected and manually analyzed. Then the principal investigator transferred the data into a computer. All responses were listed/coded based on the main questions. Similar responses were grouped together based on predefined themes as listed in Table 1. Only a couple of issues arose—husband infidelity and mistrust in laboratory results—and these were linked to the predefined themes during the analysis. The data were analyzed in the original language (Arabic) and then translated by a professional translator into English.

### Ethics approval

Ethical approval was received from the Health and Medical Ethics Committee based in the Khartoum state Ministry of Health. Furthermore, informed verbal consent was obtained from the women before the FGDs began.

## Results

### Respondents’ socio-demographic characteristics

The total number of women who participated in the FGDs was 121, of whom 72 (61 %) were pregnant (married) while the rest were of reproductive age. Most of the participants fell into the age range of 20–38 years. They represented different ethnic groups; all of them were Muslims and spoke Arabic. Most of them had basic formal education and were housewives.

### Knowledge about MTCT of HIV

Most of the women knew about the sexual transmission of HIV, but few indicated prior knowledge of the MTCT of HIV. Those who mentioned MTCT indicated that it could occur during pregnancy and via breastfeeding. No one indicated that MTCT might occur during labor. Misconceptions about transmission existed; for example, some participants believed that HIV could be transmitted by kissing, brushing teeth, communicating with people living with HIV/AIDS and mosquitoes. The majority mentioned being in a committed marriage/relationship and not having sex outside of marriage in order to prevent themselves from being infected with HIV. Interestingly, most of the women said the practice of sex outside marriage applied mainly to men; for example, a 32-year-old pregnant woman from Khartoum South said, “*I am sitting inside my house, where I could get HIV, due to my husband sleeping around.”*

### Perceived severity of HIV

The majority of the women indicated that HIV/AIDS is dangerous for women and has psychological, social and economic implications, such as depression, isolation and divorce. In contrast, a few participants indicated that it is not serious if the patient is enrolled in treatment and takes medication. For children, the consequences were perceived as being disastrous. Most of the women indicated consequences such as the death of the child either in utero or post-partum and the possibility of the child being born with disabilities. Some mentioned that the child would face stigma and be unable to complete his/her studies. “*If a child was born as HIV positive he will die very soon,”* said a 37-year-old woman in the Haj Yousif area. *“And if he lived he will be expelled from the school,”* added another 29-year-old woman in the same group.

### Perceived susceptibility of HIV

Most of the women said they were susceptible to HIV/AIDS due to Caesarean section, blood transfusion, the use of sharp items that might be contaminated or husband infidelity. *“We are sitting inside our houses and we don’t know what will happen to us”* (a 34-year-old woman in Khartoum South).

On the other hand, a few women indicated a low probability of HIV infection because they were not thinking about HIV and thereby not expecting it. They did not feel at risk of getting HIV. When it came to children, most of the women acknowledged a high probability of their children being infected by HIV and associated this with sexual abuse or rape of children and playing with sharp, contaminated items. Only a few participants linked susceptibility to the mother being infected with HIV and AIDS.

*“If a mother of a child is seeking sex outside marriage, her child will get infected by HIV and this is God’s punishment, and she deserves it”* (A 33-year-old pregnant woman in the Haj Yousif area).

### Perceived importance of HIV testing during pregnancy

Almost half of the women indicated that the HIV test is useful because it informs people whether they need to take medication, it can save the child and it gives them the opportunity to make informed decisions regarding labor and breastfeeding. It also eliminates doubts and suspicion (since some women do not trust their husbands). On the other hand, about half of the participants indicated that an HIV test is not useful because it does not have any added value: the women saw it as an additional burden of anxiety and worry.

“*It is not useful, I’m okay and I don’t need this extra burden on my shoulders”* (A 27-year-old woman in Bahri, Khartoum North).

*“If a woman knows that she hasn’t done anything wrong, then why should she go and get tested? She is not in need of troubles”* (A 35-year-old woman in Dikhinat, Khartoum South).

*“If someone (female) trusts herself and she never did anything wrong, why would she go?”* (A 28-year-old pregnant woman in Omdurman).

When asked about the usefulness of HIV testing for the child, most of the women reacted positively. In contrast, some women indicated that an HIV test is not useful for the child and were not able to recognize the risk of MTCT.

Oddly, when specifically asked about the advantages of HIV testing during pregnancy, the majority of the participants did not see any. However, some of the women mentioned advantages such as knowing their status because their husbands were not trustworthy, safeguarding the child, learning how to remain HIV-free if they tested negative and protecting the family. Some added that if they got tested, they would be relieved. One woman, a 32-year-old from Haj Yousif, said; “*so I can have an abortion and not deliver a sick child*.” Another woman, 28 years old, said, *“So I know what my status is.”*

Fear, tension, expecting non-confidentiality of test results and stigma were the main disadvantages perceived by most of the women. There was fear concerning the surrounding community, as they may suspect women who go for an HIV test to be found positive. Some women illustrated the concept of fatalism and said there are no benefits of going for a test because the disease is not curable, to which they added that they did not trust health care providers.

*“Every now and then we hear about someone who has received a laboratory result that is someone else’s”* (A 35-year-old pregnant woman in Omdurman).

### Access and influence of other people on decision to test for HIV

Most of the women mentioned the doctor as the most influential person regarding the decision to test, followed by the pregnant woman herself, her husband, her parents and her elder brother. Mothers-in-law and friends also influenced decisions related to HIV testing. In general, they said that if the doctor requests a test, they will do it. Involvement of husbands was also perceived as positive. However, most of the women claimed that no one makes decisions on their behalf. *“Hey, I am the only one who decides on this, no one can force me”* (A 27-year-old pregnant woman in Khartoum South).

For factors affecting the access and acceptance of HIV testing during pregnancy, the majority of the women mentioned health-facility-based factors, including the distance to the testing center, the duration of the test, non-confidential behavior and the HIV test being made routine and compulsory. Non-health-facility factors included involvement of husbands and awareness of the benefits of the test, which were mentioned by some of the women.

### Perceived norm-violating behavior and stigma

According to the majority of women, a mother is not responsible for becoming infected with HIV, and if she is infected, that means she got it from her husband. On the other hand, some indicated that a woman and her husband have joint responsibility. *“As the husband sleeps around, the wife does too”* (A 36-year-old woman in Khartoum North).

However, a few women indicated that a mother is responsible if she behaves in a way that violates norms, like having sex outside her marriage.

*“The wife whose husband is working abroad and who hasn’t managed to abstain from sex might get infected, and thereby pass it to the child”* (A 28-year-old woman in the Haj Yusuf area).

Some women indicated that in Sudan, HIV-positive persons are perceived to be bad persons who are guilty and have committed sins. People tend to forget about other modes of transmission besides sexual transmission and are inclined to attribute infection to the person’s own behavior. This blaming seems to result in stigmatization. Almost all women stated that children are not responsible for getting HIV because they are new to this world and do not choose their parents. When we asked about behavior related to becoming infected with HIV, most women mentioned illegal sex, meaning sex outside marriage.

The answers to the question of whether an HIV-positive person should be allowed to work in a public place were twofold. While half of the participants said “Yes” while hinting that the HIV-positive person must not have direct contact with other people, the other half said “No, s/he must not be allowed to work at all, because s/he will infect others.” *“I can’t buy food from someone who is infected with HIV/AIDS,”* said one 37-year-old woman in Khartoum North.

In terms of disclosure, the majority of women stated that an HIV-positive person should not let others know his/her status, because s/he will get stigmatized (e.g., s/he could lose his/her job and be forced to leave the family home). Even those who supported the disclosure of HIV status qualified their statements by saying that, in order for others to protect themselves, they should not have contact with an infected person. Very few participants said they would inform relatives who could help them.

## Discussion

This study revealed that Sudanese women of reproductive age are aware of the sexual transmission of HIV, but they are not aware of the risk of MTCT, especially during labor. This conforms to findings in a study in rural southern India [25] in which knowledge about MTCT was low, as well as to a further study in Nigeria [17]. On the other hand, it contradicts findings from Botswana [26], Ethiopia [27] and Nigeria [17], which indicated a high level of MTCT knowledge among pregnant women. This could be attributed to the fact that PMTCT services are quite new in Sudan compared to those in Nigeria and Botswana.

Our study showed that most women perceived themselves to be at risk of and susceptible to HIV infection. This contradicts findings from a study in India [25] in which 97 % of women did not perceive themselves to be at risk of acquiring HIV. However, in our study this perception was not associated with a motivation for HIV testing, as suggested by Maman et al. [28], who found that among Tanzanian women the perception of personal susceptibility to HIV infection serves as a primary factor for motivating women to get an HIV test. It seems that the women in our study were not motivated to get tested to know their HIV status. A husband’s infidelity was found to be a real concern among women who believe that they feel at risk of HIV for this reason; this contrasted with a study in Kenya [29] in which a husband’s HIV/AIDS infection was attributed to his female partner’s promiscuous and unfaithful behavior.

In our study, we found that women will accept an HIV test if it is offered by a doctor. Despite other factors viewed as barriers (e.g., distance and time spent taking the test), this emphasizes the great influence that doctors and health cadres in Sudan have on the decisions made by pregnant women.

Our study also revealed that the HIV test is perceived as useful by some women for themselves and their unborn children; these perceived benefits of HIV testing were in line with findings from pregnant women in Tanzania [28].

Health-facility-related factors such as access, confidentiality, time spent at an antenatal clinic (ANC) and mistrust of laboratory results were mentioned as being among key issues affecting the intention to go for HIV testing. The first three factors fit in with findings from India [30] and with the findings of the systematic review by Colvin J. Christopher et al. [13], while the mistrust in laboratory results conforms to findings from Uganda [31].

Stigma was among the key factors interlinked with the intention and decision to have an HIV test performed during pregnancy. We also found that the majority of women were not willing to disclose their HIV status to anyone. The main perceived consequences of disclosure are stigma and the resultant expectation of job loss and being forced to leave the family. This is in line with findings by Turan M J et al. [21] and Maman et al. in Tanzania [28] in which fear of conflict with partners was mentioned as a main factor for non-disclosure. On the other hand, our findings contrast with conclusions of a study in Botswana [20] in which most of the women (85 %) had disclosed their HIV status, most commonly to their partners. However, in both studies, the consequences are similar and the end result is stigma and discrimination.

Our study had a reasonable sample size despite the sensitivity of the topic and the uniqueness of the respondents. A limitation of this study is that it was conducted among women in Khartoum (and peripheral neighborhoods) only, which might endanger the generalizability of the findings. However, as the capital city, Khartoum typically represents a wide spectrum of people originating from all other regions of Sudan. The use of notes instead of tapes could also be a limitation; however, this resulted in a lot of advantages in getting genuine information from the women, who participated freely.

## Conclusions

In summary, our analysis reveals that if doctors offer the test, pregnant women will accept it. Duration of the test and confidentiality were also mentioned among other health-facility-related factors that influence the decision to undergo the test. Involvement of husbands and awareness of the benefits of the test were mentioned, but with less emphasis than the above factors. Because of anticipated stigma, almost all of the women mentioned that they would never disclose their HIV status if they tested positive. It is recommended that for any HIV/AIDS program, a holistic approach that tackles all aspects of the program should be adopted. In addition, the adequate implementation of PITC should be critically assessed in future research about PMTCT in Sudan.

## Abbreviations

AIDS: Acquired immune deficiency syndrome
ANC: Antenatal Clinic
ARV: Antiretrovirals
CD4: Cluster of differentiation
FGDs: Focus group discussions
HIV: Human immunodeficiency virus
MENA: Middle East and North Africa
MTCT: Mother to child transmission
NGO: Non-Governmental Organization
PITC: Provider initiated testing and counseling
PMTCT: Prevention of mother to child transmission
